# Sprouty2/4 deficiency disrupts early signaling centers impacting chondrogenesis in the mouse forelimb

**DOI:** 10.1093/jbmrpl/ziaf002

**Published:** 2025-01-10

**Authors:** Linda Dalecka, Eva Hruba, Marketa Andrasova, Klara Steklikova, Zuzana Pavlikova, Klara Kucerova, Tereza Szotkowska, Martin Bartos, Marcela Buchtova, Abigail Saffron Tucker, Maria Hovorakova

**Affiliations:** First Faculty of Medicine, Institute of Histology and Embryology, Charles University, 128 00 Prague, Czech Republic; Department of Cell Biology, Faculty of Science, Charles University, 128 00 Prague, Czech Republic; Institute of Animal Physiology and Genetics, Czech Academy of Sciences, 602 00 Brno, Czech Republic; Department of Experimental Biology, Faculty of Science, Masaryk University, 625 00 Brno, Czech Republic; First Faculty of Medicine, Institute of Histology and Embryology, Charles University, 128 00 Prague, Czech Republic; Department of Anthropology and Human Genetics, Faculty of Science, Charles University, 128 00 Prague, Czech Republic; First Faculty of Medicine, Institute of Histology and Embryology, Charles University, 128 00 Prague, Czech Republic; Department of Cell Biology, Faculty of Science, Charles University, 128 00 Prague, Czech Republic; First Faculty of Medicine, Institute of Histology and Embryology, Charles University, 128 00 Prague, Czech Republic; Department of Anthropology and Human Genetics, Faculty of Science, Charles University, 128 00 Prague, Czech Republic; Institute of Animal Physiology and Genetics, Czech Academy of Sciences, 602 00 Brno, Czech Republic; Department of Experimental Biology, Faculty of Science, Masaryk University, 625 00 Brno, Czech Republic; Institute of Animal Physiology and Genetics, Czech Academy of Sciences, 602 00 Brno, Czech Republic; First Faculty of Medicine, General University Hospital, Institute of Dental Medicine, 121 08 Prague, Czech Republic; First Faculty of Medicine, Institute of Anatomy, Charles University, 128 00 Prague, Czech Republic; Institute of Animal Physiology and Genetics, Czech Academy of Sciences, 602 00 Brno, Czech Republic; Department of Experimental Biology, Faculty of Science, Masaryk University, 625 00 Brno, Czech Republic; First Faculty of Medicine, Institute of Histology and Embryology, Charles University, 128 00 Prague, Czech Republic; Centre for Craniofacial and Regenerative Biology, Faculty of Dentistry, Oral and Craniofacial Sciences, King’s College London, Guys Hospital, London, TN8 7LR, United Kingdom; First Faculty of Medicine, Institute of Histology and Embryology, Charles University, 128 00 Prague, Czech Republic

**Keywords:** FGF signaling, RTKs, micro-CT, autopodium pathologies, apical ectodermal ridge, zone of polarizing activity, Shh, ciliopathy, limb patterning, genetic animal models

## Abstract

The FGF signaling pathway plays an important role in the regulation of limb development, controlling cell migration, proliferation, differentiation, and apoptosis. Sprouty proteins act as antagonists of the FGF pathway and control the extent of FGF signaling as part of a negative feedback loop. *Sprouty2/4* deficient mice evince defects in endochondral bone formation and digit patterning in their forelimbs, with pathogenesis recently related to ciliopathies. To understand the mechanisms behind these pathologies, the limb defects in *Sprouty2+/−;Sprouty4−/−* male and female mice were characterized and correlated to the dynamic expression patterns of *Sprouty2* and *Sprouty4*, and the impact on the main signaling centers of the limb bud was assessed. *Sprouty2* and *Sprouty4* exhibited dynamic expressions during limb development. Interestingly, despite similar expression patterns in all limbs, the hindlimbs did not evince any obvious alterations in development, while the forelimbs showed consistent phenotypes of variable severity. Prenatally as well as postnatally, the left forelimb was significantly more severely affected than the right one. A broad variety of pathologies was present in the autopodium of the forelimb, including changes in digit number, size, shape, and number of bones, hand clefts, and digit fusions. Ectopic ossification of bones and abnormal bone fusions detected in micro-CT scans were frequently observed in the digital as well as in the carpal and metacarpal areas. *Sprouty2+/−;Sprouty4−/−* limb buds showed patchy loss of *Fgf8* expression in the apical ectodermal ridge, and a loss of tissue underlying these regions. The zone of polarizing activity was also impacted, with lineage analysis highlighting a change in the contribution of Sonic hedgehog expressing cells. These findings support the link between Sproutys and Hedgehog signaling during limb development and highlight the importance of Sprouty2 and Sprouty4 in controlling early signaling centers in the limb.

## Graphical Abstract

**Graphical Abstract:**
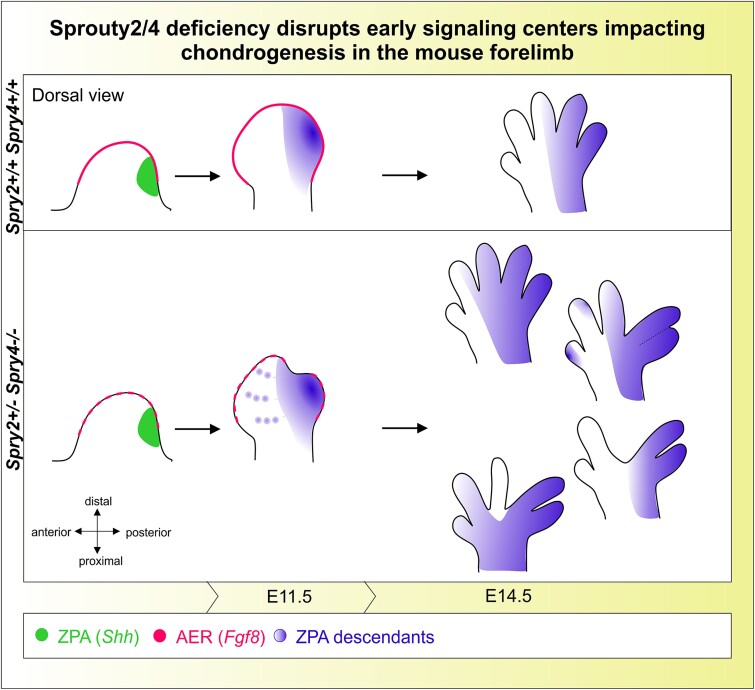

## Introduction

The FGF signaling pathway plays a key role in the regulation of cell migration, proliferation, differentiation, and apoptosis, and is crucial for correct limb development. Expression of *Fgf10* in the lateral plate mesoderm is essential for initiation of the limb bud.^1^^,^^2^ During early steps of limb bud formation, Fgf10 interacts with Fgf8 originating in the apical ectodermal ridge (AER)^3^ and Sonic hedgehog (*Shh*) from the zone of polarizing activity (ZPA) through Fgfr2.^1^^,^^4–6^

Sprouty proteins are a family of FGF antagonists described originally in the Drosophila airways.^7^ Sprouty homologs have been found in vertebrates, with 4 *Sprouty* genes: *Spry1*, *2*, *3*, and *4* identified in the mouse and human genomes.^8^^,^^9^ Sprouty proteins work as inhibitors of receptor tyrosine kinase pathways (RTKs) and participate in negative feedback loop interactions during mammalian development.^10^ Sprouty1, 2, and 4 are expressed in the developing brain, teeth, palate, salivary glands, lungs, and kidneys, participating in the regulation of epithelial-mesenchymal interactions during developmental processes^eg.,^.^11^  ^12^ In the limb, Sprouty2 protein expression has been detected in the mesenchyme of the chick limb buds at embryonic day (E) 3.5 outside the AER.^13^ In the mouse, the expression of Sprouty1, 2, and 4 were observed in the forming limb bud mesenchyme from E9.5 to E11.5.^8^^,^^14^ Similar to the chick, murine Sprouty4 was limited to mesenchymal cells adjacent to the AER, excluding the AER itself at E11.5.^8^ During later limb development, *Sprouty1* and *2* expression was associated with forming muscles and tendons.^15^  *Sprouty* genes, therefore, have overlapping expression at several stages of limb development.

Mice lacking normal levels of Sprouty2 and/or 4 exhibit pathologies in the craniofacial region, lungs, and limbs.^16^^,^^17^  *Sprouty2* has been shown to be a regulator of endochondral ossification and to regulate proliferation and differentiation of chondrocytes.^18^ The pathogenesis in *Sprouty* deficient animals has been recently related to the abnormal formation of primary cilia.^19^ The ciliary axonemes in embryonic and postnatal growth plate chondrocytes were elongated in *Sprouty2/4* deficient mice. In keeping with this, cilia-dependent Hedgehog signaling was shown to be upregulated in *Sprouty2/4* deficient animals.^19^ The relationship of *Sprouty* and the Shh pathway has also been suggested in teeth, where Sprouty2 and 4 deficiencies led to disrupted Shh expression and consequent formation of supernumeraries in the antemolar area.^17^

In the limb, Shh plays a key developmental role via its expression in the ZPA, which specifies the antero-posterior axis of the autopodium.^4^^,^^5^  *Shh* expression in the polarizing region is also controlled by FGF signaling from the AER. FGF signaling has been shown to regulate the expression of the transcription factors ETV4 and ETV5, which suppress *Shh* expression outside of the ZPA.^20^

Interestingly, cell lineage tracing experiments determining the fate of the Shh expressing cells of the ZPA have shown that the 2 posterior digits of the mouse limb derived entirely from the ZPA with some contribution of cells to the third digit. The 2 anterior digits were shown to originate outside the polarizing zone.^21^ According to recent limb patterning model, the AER of the mouse limb completely overlies the polarizing region, enabling the cells of the ZPA to expand and give rise to the more posterior digits by self-organization.^22^

Ectopic expression of *Shh* at the anterior margin of the limb in mouse mutants led to polydactyly.^5^ In contrast, in mouse embryos lacking Shh only one digit-like structure was formed in the hindlimbs, and no digits were present in the forelimbs.^23^ Temporal loss of *Shh* confirmed that fewer digits developed when *Shh* was deleted at earlier stages,^24^ suggesting that *Shh* plays different roles in digit patterning dependent on developmental stage. In the first phase, *Shh* specifies positional values across the antero-posterior axis of the very early limb bud, while in the second phase *Shh* is required to support proliferation and survival of cells that will form the digits.^24^

Although limb pathologies have been reported in *Sprouty* mutants, a full analysis of the types of defects observed and their penetrance has not been previously reported. In this article, we have focused on *Spry2+/−;Spry4−/−* animals. *Spry2−/−* are lethal postnatally. The double knockouts are embryonic lethal, while *Spry2+/−* mice have limited limb phenotypes.^19^ In contrast, *Spry2+/−*;*Spry4−/−* are viable, allowing detailed analysis of their limbs postnatally. Limbs of these mice have already been shown to have digit patterning defects at E17.5, while chondroma-like nodules were observed on the ribs and long bones.^19^

For our analysis, limbs of *Spry2+/−;Spry4−/−* adult mice were analyzed by micro-CT (*μ*CT) and Alcian Blue and Alizarin Red staining of skeletal structures, followed by an investigation of the mechanisms that led to the observed limb abnormalities. The research highlights that Sproutys play multiple roles during limb formation, with dynamic expression patterns during limb development. Loss of Sproutys impacted both the AER and ZPA during early patterning, and chondrogenesis in later development, leading to complex final limb defects. This appreciation of the complexity and underlying mechanisms involved in *Spry2+/−;Spry4−/−* limb defects can help explain the often variable and complex limb defects observed in patients with ciliopathies.

## Materials and methods

### Mice

*Spry2+/−;Spry4+/−* mice were crossed with *B6.Cg-Shhtm1(EGFP/cre)Cjt/J* (Jackson Laboratory) and *B6.129S4-Gt(ROSA)26Sortm1LacZSor/J* (Jackson Laboratory) strains, respectively, in order to obtain mice for further breeding. All the used animals were kept on the same genetic background *B6*.

Heterozygotes for *Spry2* and *Spry4* alleles in the offspring did not evince any pathologies and were viable and fertile. *Spry2+/−;Spry4+/−/B6.129S4-Gt(ROSA)26Sortm1LacZSor/J* breeding pairs were used to generate *Spry2+/−;Spry4−/−* and control *Spry2+/+;Spry4+/+* specimens used for analysis. For lineage tracing experiments, heterozygote males *Spry2+/−;Spry4+/−/B6.Cg-Shhtm1(EGFP/cre)Cjt/J* were mated with heterozygote females *Spry2+/−;Spry4+/−/B6.129S4-Gt(ROSA)26Sortm1LacZSor/J* in order to obtain embryos/fetuses allowing visualization of cells expressing Shh and their offspring using X-gal staining.

Mice and prenatal specimens were genotyped using a standard protocol (Jackson Laboratory) under standard PCR conditions with an annealing temperature of 50 °C for the *Sprouty2* gene and 57 °C for the *Sprouty4* gene. Primers used for genotyping *Sprouty2* were as follows: 5′-GCATGGGCTATTCACAAAC-3′; 5′-TTGAGAACATGCCTCGACC-3′; and 5′-GGATGGCTCTGATCTGATCC-3′. Primers used to genotype *Sprouty4* were as follows: 5′-CAGGACTTGGGAGTGCTTCCTTAG-3′; 5′-CCTCCTAGTACCTTTTTGGGGAGA G -3′; and 5′-TACAGCAGGAATGGCTACGGTG-3′.

*Spry2+/−;Spry4−/−* embryos/fetuses were harvested between E12.5 and 18.5 (*n* = 40). Percentage of *Spry2+/−;Spry4−/−* at different harvesting days varied from 7% to 26% (Table 1).

**Table 1:** Numbers and percentages of prenatal specimens for each genotype harvested at different time points.

Collected stage	E12.5	E13.5	E14.5	E15.5	E16.5	E17.5	E18.5	In total
**Harvested specimen**	70	42	29	35	21	19	39	255
**Genotype *S2+/−S4−/−***	11	3	4	8	3	5	6	40
**Percentage (%)**	16	7	14	23	14	26	15	16
**Genotype *S2+/+S4+/+***	12	4	7	2	3	2	5	35
**Percentage (%)**	17	10	24	6	14	11	13	14
**Genotype *S2+/*−*S4+/*−**	13	10	10	8	6	5	11	63
**Percentage (%)**	19	24	34	23	29	26	28	25
**Genotype *S2+/+S4+/*−**	9	11	3	7	6	6	9	51
**Percentage (%)**	13	26	10	20	29	32	23	20
**Genotype *S2+/+S4*−*/*−**	16	5	1	3	0	0	2	27
**Percentage (%)**	23	12	3	9	0	0	5	11
**Genotype *S2+/*−*S4+/+***	8	9	2	6	3	1	5	34
**Percentage (%)**	11	21	7	17	14	5	13	13
**Genotype *S2*−*/*−*S4+/*−**	1	0	1	0	0	0	1	3
**Percentage (%)**	1	0	3	0	0	0	3	1
**Genotype *S2*−*/*−*S4*−*/*−**	0	0	1	1	0	0	0	2
**Percentage (%)**	0	0	3	3	0	0	0	1

Abbreviations: E, embryonic day; S, Sprouty.

Mice were mated overnight, with midnight before the morning detection of the vaginal plug determined as E0.0. Pregnant mice were euthanized by cervical dislocation. Immediately after removing the embryos, their wet body weight (BW) was determined to refine the chronological staging, with BW correlating to developmental stage.^25^ Prenatal wild type (WT) mice (CD1and B6 background strains) were analyzed by RNAscope (E11.5-13.5), and whole mount *in situ* hybridization (WISH) (E10.5-11.5 for *Fgf8*, E12.5-16.5 for *Sprouty2*).

### Whole mount *in situ* hybridization

Whole mount *in situ* hybridizations were performed using standard methods.^26^ Forelimbs from *Spry2+/−;Spry4−/−* and littermate controls were dissected and fixed in 4% paraformaldehyde (PFA) at E10.5-11.5. Tissues were dehydrated and rehydrated through a methanol series, treated with Proteinase K (10 μg/mL) for 30 min at room temperature, and postfixed in 4% PFA/25% glutaraldehyde solution for 20 min at RT. Tissues were incubated with *Fgf8* digoxygenin labeled probe in a hybridization mix at 68 °C overnight while rotating in a hybridization oven (Compact Line OV4). Subsequently, the tissues were incubated with antidigoxigenin conjugated to alkaline phosphatase (AP; 11 093 274 910, Roche, Switzerland) overnight at 4 °C while shaking. The signal was developed using BM Purple (11442074001). Pictures were captured using a Leica M205 FA stereoscope.

For detection of *Sprouty2* gene expression, WT embryos (E12.5-16.5) were fixed in 4% PFA for 24 hr and washed in PBS and stored in methanol. After rehydration, the limbs were hybridized with *Sprouty2* probe prepared from plasmid 3ME6/mSprouty2 pBSKS.

### Gene expression analyses by RNAscope

RNAscope (Advanced Cell Diagnostics) detection of *Sprouty2* and *Sprouty4* mRNA was performed in WT (CD1 and B6) control embryos using the RNAscope Multiplex Fluorescent v2 Kit following the manufacturer’s recommendations. The RNAscope method was applied to paraffin sections of tissue using validated probes for *Sprouty2* and *Sprouty4* (Cat. No. 425061, Cat. No. 417411-C3). In order to detect a fluorescent signal, Fluorescein (FP1168) and Cyanine3 (FP1170) were applied from the TSA Plus fluorescein system diluted in TSA buffer (322809) at a ration of 1:1000. Images were captured on Leica SP8 Confocal microscope and processed using the LasX software program.

### Gene expression analyses by qPCR

Limb tissues (*n* = 25, E11.5 to E13.5) of Sprouty deficient and control embryos were lysed in RLT buffer, followed by total RNA purification using the RNeasy Plus Mini Kit (QIAGEN, Denmark). Reverse transcription reaction was performed using the gb Elite Reverse Transcription kit (Generi Biotech, Czech Republic). Subsequent qPCR was carried out using the gb Ideal PCR Master Mix (Generi Biotech, Czech Republic). The expression levels of *Gremlin1* (*Grem1*) and *CyclinD1* (*CycD1*) were analyzed using TaqMan Gene Expression Assays (ID: Mm00488615_s1 and Mm00432359_m1). *β-actin* served as a housekeeping control (Mm00607939_s1) (Thermo Fischer Scientific, USA).

### *Shh* expressing cell-lineages tracing

*Shh* expressing cell lineage was traced using X-gal staining in the offspring of *Spry2+/−;Spry4+/−/B6.129S4-Gt(ROSA)26Sortm1LacZSor/J* females crossed with *Spry2+/−;Spry4+/−/B6.Cg-Shhtm1(EGFP/cre)Cjt/J* males.

*Spry2+/−;Spry4−/−* embryos (*n* = 7) and their littermates were harvested at E14.5. Dissected embryonic limbs were washed in a phosphate buffer at 4 °C and prefixed for 20 min in 4% PFA, before staining in X-gal (Sigma) (concentration 3 mM). Beta-galactosidase activity was detected by incubation in the dark O/N at 37 °C. Samples were postfixed in 4% PFA O/N and washed in PBS. Limbs were photographed using a Leica MZ16 stereomicroscope (Leica Microsystems GmbH) equipped with a Zeiss Axiocam 208 color digital camera (Zeiss).

### Postnatal analysis and micro-CT (*μ*CT)

*Spry2+/−;Spry4−/−* specimens (*n*_spec_ = 20, *n*_limbs_ = 40, *n*_males_ = 9, *n*_females_ = 11) (aged from 5 to 33 wk) were dissected and fixed in 4% PFA O/N 4 °C. Samples were washed in PBS and documented using a Leica MZ16 stereomicroscope (Leica Microsystems GmbH) equipped with a Zeiss Axiocam 208 color digital camera (Zeiss).

Samples in PBS were scanned ex vivo using a *μ*CT desktop device SkyScan 1272 (Bruker micro-CT) with following parameters: pixel size of 15 μm, source voltage 70 kV, source current 142 μA, 0.5 mm Al filter, rotation step = 0.6°, rotation of 180°, scanning time was approximately 20 min per specimen. Projection images were reconstructed using NRecon software (Bruker). Visualizations were acquired in standardized setting using DataViewer for 2D imaging and CTVox software (Bruker) for 3D imaging.

### Histology

Selected representative samples of *Spry2+/−;Spry4−/−* forelimbs fixed in 4% PFA after *μ*CT scanning were decalcified in Biodec-R (Bio-Optica). Decalcified samples were embedded in paraffin and 7 μm sections were prepared using a Leica RM2245 semi-automated rotary microtome (Leica Microsystems GmbH). Sections were stained with Masson’s green trichrome, dehydrated, and covered using DPX mountant for histology (Sigma-Aldrich). The sections were documented using DMLB Leica microscope equipped with a Leica MC170 HD camera (Leica Microsystems GmbH).

### Cartilage and bone staining

Selected *Spry2+/−;Spry4−/−* postnatal samples were prepared for clearing by removing skin and adipose tissue followed by dehydration to 100% ethanol. Samples were stained with 0.03% Alcian Blue solution (cartilage staining), washed in 70% ethanol, and incubated in 96% ethanol overnight. Ethanol was replaced with 1% potassium hydroxide (KOH) to preclear the specimens. Samples were consequently stained with 0.001% Alizarin Red solution (bone staining). Finally, the samples were placed into a 1% KOH clearing solution, which was replaced with 100% glycerol and 1% KOH (1:1). For long-term storage, the samples were placed in 100% glycerol.

### Statistical analysis

**Figure 1:**
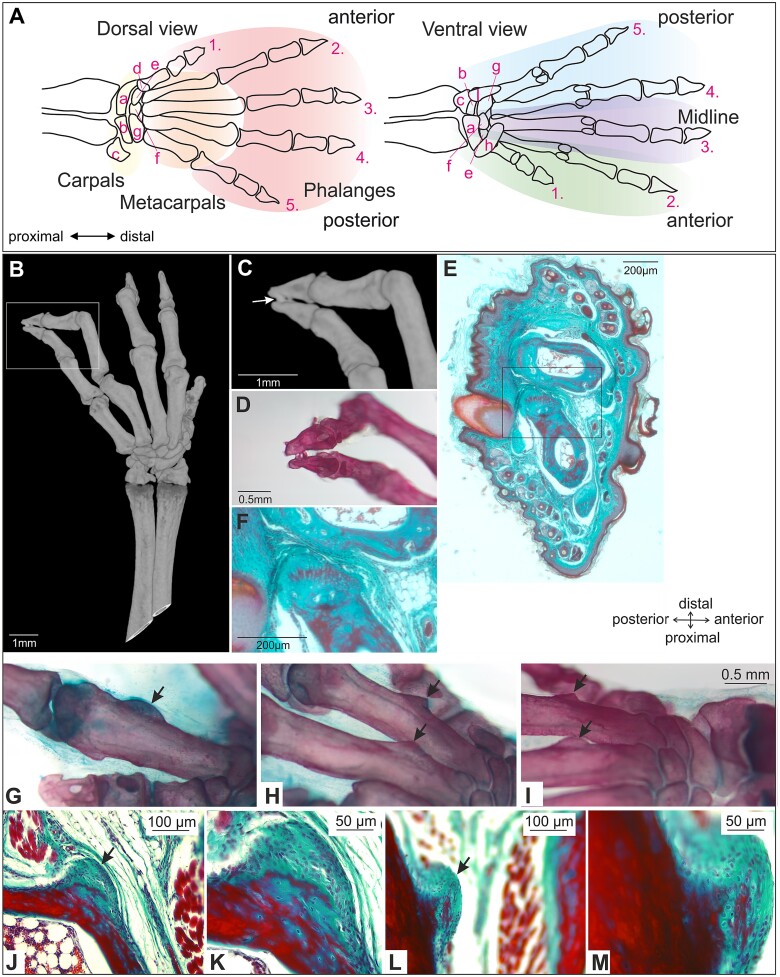
Schematic showing the areas of the forelimb autopodium analyzed and bone defects in *Spry2+/−;Spry4−/−* mice. (A) Dorsal view: the carpal bones (a-g), metacarpal bones, and phalangeal bones are visualised schematically. Ventral view: the anterior-posterior regions are visualized schematically. (B, C) Synostosis of the distal phalanges (arrow) in the left limb of a 6 weeks old *Spry2+/−;Spry4−/−* male shown in μCT, and (D) by using Alizarin Red (bone) and Alcian Blue (cartilage). (E, F) Histology of the same specimen. Area of the insert in B is shown in detail in C. Insert in E is shown in F in detail. (G-I) Nodules (arrows) on bones of autopodium of *Spry2+/−;Spry4−/−* adult mice stained with Alizarin Red (bone) and Alcian Blue (cartilage). (J-M) Histological sections stained with Masson’s green trichrome. Ectopic areas of ossification were frequently detected on the bones in the autopodium in *Spry2+/−;Spry4−/−* adult mice. These structures evinced a higher degree of ossification as shown by both: Alizarin Red wholemount and trichrome stained sectioned autopodia of *Spry2+/−;Spry4−/−* mice. K and M show a higher magnification of J and L, respectively.

Scanned limbs were categorized based on defect: syndactyly, oligodactyly, camptodactyly, cleft etc and area (carpal bones, metacarpal bones, phalangeal bones, anterior segment of the autopodium, midline, posterior segment of the autopodium; see also the Figures 1A, S1, and S2). A Mann–Whitney nonparametric *U*-test was used to test the statistical dependence or independence of the severity of the defects ($Z=\frac{U-\mu +c}{6}$). The number of affected bones in each limb was the main variable taken into consideration and its dependence on visible phenotype was tested. Chi-square test $\Big({x}^2=\sum_{i=1}^n\frac{{\left({o}_i-{e}_i\right)}^2}{e_i}\Big)$ was used to test the relationship between the regions of defects comparing the number of affected limbs in each region (anterior/midline/posterior, carpal/metacarpal/phalangeal). For testing, the statistical dependence between the severity of the defect represented by the number of affected bones and laterality in both sexes a Wilcoxon signed-rank test was used $\left(Z=\frac{W-\mu +C}{6}\right)$.

### Ethical statement

All experiments were carried out in strict accordance with the protocol approved by the Expert committees for the guarantee of good life conditions of experimental animals at First Medical Faculty at Charles University, Prague, Czech Republic (permit number: MSMT-272/2020-3).

## Results

### Micro-CT reveals hidden limb phenotypes in *Spry2+/−;Spry4−/−*

To map the prevalence and severity of the limb defects in *Spry2+/−;Spry4−/−* mice, forelimbs and hindlimbs were analyzed in adult animals (average age 8 wk), using *μ*CT scans. Hindlimbs developed normally in all harvested specimens (data not shown), while the forelimbs displayed a range of defects of the autopodium (hand) (Figures S1 and S2). Interestingly, *μ*CT revealed a number of autopod pathologies not obvious by visual inspection, such as defects in sesamoid bones and ectopic mineralized nodules, highlighting the importance of using this technique. *μ*CT analysis revealed defects in 95% of *Spry2+/−;Spry4−/−* mice (19 out of 20 specimens, 37 forelimbs out of 40), compared to 55% analyzed visually (11 out of 20 specimens). As expected, the total number of affected bones of the autopodium was significantly associated with the presence of a visible phenotype (Mann–Whitney nonparametrical test, *p*-value = .00001797). Thus, if the phenotype was detectable also macroscopically and without *μ*CT examination, the limb was more seriously affected with a higher number of defective bones.

### Major autopodium pathologies were detected in the middle carpal area in *Spry2+/−;Spry4−/−* adult animals

*μ*CT scan analysis revealed a relatively broad spectrum of pathologies of the forelimb in *Spry2+/−;Spry4−/−* adult animals differing in their position and severity (Tables 2-4, Figures S1 and S2). Synostosis (fusion of bones) was detected in 33 out of 37 harvested affected forelimbs and was the most frequently observed pathology (89%). Oligodactyly (fewer than 5 digits) was observed in 7 forelimbs of 7 different specimens (19% of all harvested affected forelimbs), while syndactyly (fusion of digits) was observed in 8 forelimbs of 6 different specimens (22% of all harvested affected forelimbs). The syndactylies included both: syndactylies caused by synostoses and by the fusions of only soft tissues (Table 2, Figures 1B-F, S1, and S2). Camptodactyly (bent digits) was observed in 15 forelimbs of 11 specimens (41% of all harvested affected forelimbs). Abnormalities of sesamoid bones were relatively common, observed in 21 forelimbs of 14 specimens (57% of all harvested affected forelimbs). A pronounced cleft of the autopodium was present in a total of 14 forelimbs of 9 specimens (38% of all harvested affected forelimbs). Interestingly, 86% of limbs had a detectable defect in their midline (Table 3, Figures S1 and S2). Numerous ectopic mineralized nodules were observed on the surfaces of autopodium bones in *Spry2+/−;Spry4−/−* adult mice. These structures showed a distinct degree of mineralization, ossifying from the superficial areas of affected bones (Figure 1G-M). Interestingly, these nodules were located along the metacarpal area in the majority of cases.

**Table 2:** Quantification of pathologies detected postnatally in *Spry2+/*−*;Spry4*−*/*−.

Types of defects		
Defect	Affected limbs, *n*	Percentage, %
**Total**	37	100
**Syndactyly**	8	22
**Oligodactyly**	7	19
**Camptodactyly**	15	41
**Sezamoids**	21	57
**Cleft**	14	38
**Nodules**	11	30
**Synostosis**	33	89

**Table 3:** Distribution of forelimb pathologies in the autopodium in *Spry2+/−;Spry4−/−* postnatal specimens.

Areas of defects		
Area of the defect	Affected limbs, *n*	Percentage, %
**Carpals**	35	95
**Metacarpals**	20	54
**Phalanges**	25	68
**Posterior**	22	59
**Midline**	32	86
**Anterior**	27	73

**Table 4:** Distribution of pathologies in the carpal, metacarpal, and phalangeal regions in postnatal *Spry2+/−;Spry4−/−*.

	Carpal bones defects		Metacarpal bones defects		Phalangeal bones defects	
Area of the defect	Affected limbs, *n*	Percentage, %	Affected limbs, *n*	Percentage, %	Affected limbs, *n*	Percentage, %
**Posterior**	9	26	12	60	19	76
**Midline**	29	83	12	60	13	52
**Anterior**	24	69	15	75	14	56

**Figure 2:**
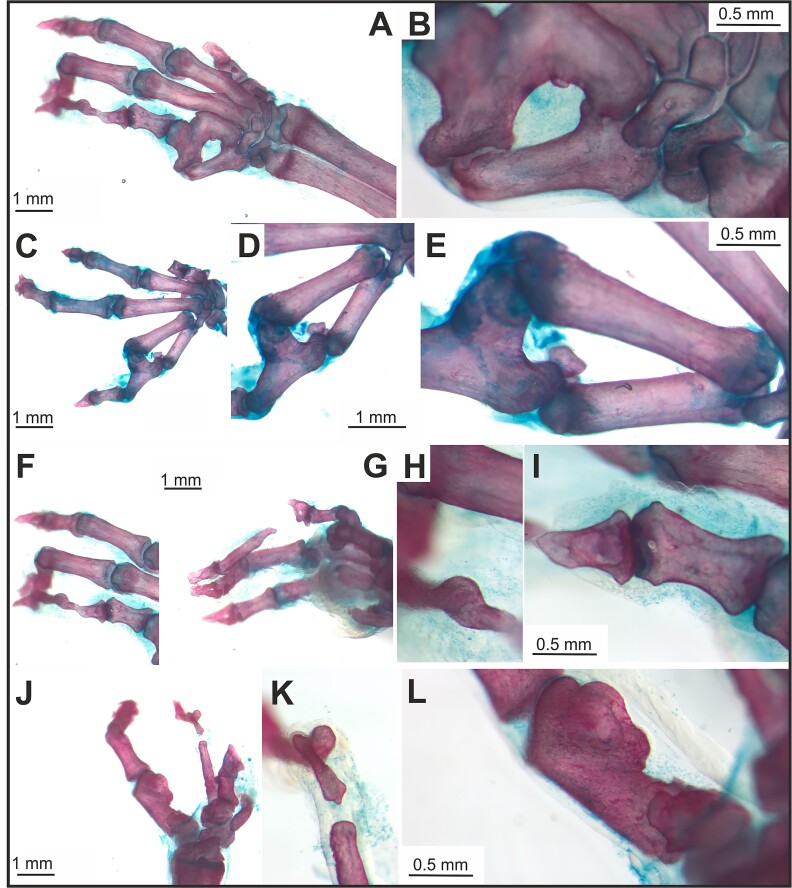
Bone defects of the autopodium in *Spry2+/−;Spry4−/−* adult mice visualized with Alizarin Red (bone ) and Alcian Blue (cartilage). Variable synostoses were detectable in the metacarpal (A, B) and proximal phalangeal (C-E) areas of the anterior autopodium. (F-I) Phalanges of neighboring fingers were fused in their distal areas with soft tissues, while the proximal phalanx of one of the fused fingers was absent. (F) Dorsal view. (G) Ventral view on the same autopodium. (H, I) Detail of a finger shown in (F). (J, K) Malformed phalanx separated by soft tissue from a malformed metacarpal bone. (L) Detail of a malformed metacarpal bone (J) with ossified structure on its distal end.

All parts of the autopodium (carpal/metacarpal/phalangeal) were affected by pathologies (Tables 3 and 4, Figures 1 and 2). Excluding the presence of isolated sesamoid bone defects, pathologies were located in the carpal bones (in 35 limbs of 19 specimens, 95% of all harvested affected forelimbs), metacarpal bones (in 20 limbs of 14 specimens, 54% of all harvested affected forelimbs) as well as in the phalangeal area (in 25 limbs of 17 specimens, 68% of all harvested affected forelimbs). 43% of all harvested limbs (16 limbs) evinced a pathology in all 3 areas. In the carpal area, the most affected part of the limb was the midline region (83%). In the metacarpal area, mainly the anterior segment was affected (75%). In contrast, in the phalangeal area, it was the posterior segment (76%). Overall, the location of these pathologies (antero-posterior vs proximo-distal) was not random (Chi-square test, *p* value = .036995), suggesting that *Sprouty* genes play several roles during the development and patterning of the forelimb.

#### Phalangeal area of the autopodium

Of the limbs with pathologies, 67% showed a defect in the phalangeal area. All areas of the phalangeal region were affected, the posterior phalangeal area (76% of all limbs with defects in the phalangeal area), the anterior phalangeal area (56%) as well as the midline finger (52%) (Table 4; Figures 1, S1, and S2). Variable synostoses were detectable between the metacarpal bones and proximal phalangeal areas (Figures 2C-E, S1, and S2). Hard and soft tissue fusions of neighboring phalanges were observed in the distal region (Figure 1B-F), as well as the absence of proximal phalanges in one of the fused fingers (Figures 2G, S1, and S2).

#### Metacarpal area of the autopodium

Similarly to the phalangeal area, all segments of the metacarpal region were affected, the anterior segment in 75% and the posterior segment and the midline in 60% of limbs with pathologies (Table 4; Figures 1G-M and 2A, B).

#### Carpal area of the autopodium

Carpal bones were affected in 95% of limbs with pathologies and interestingly, carpal defects frequently included carpal bones synostosis (26 out of 35 forelimbs affected in their carpal area, including both: synostosis between carpal bones and/or synostosis between carpal and metacarpal bones). In the carpal region, different abnormal shapes or sizes of carpal bones, supernumerary bones, ectopic ossification and carpal bone reorganizations were observed (Figures 1, 2, S1, and S2). 24% (9 limbs) of all harvested limbs had pathologies present only in the carpal area. Similarly to metacarpal area, the carpal bones were more frequently and also more seriously affected on the anterior side of the autopodium (69%) in contrast to the posterior side (26% of carpal bones). However, the majority of pathologies in the carpal area were located in the midline (83%) (Table 4).

### Laterality and sex play a role in severity of the defect in the forelimb

Interestingly, the left limbs of *Spry2+/−;Spry4−/−* animals were more severely affected than the right limbs, with 14/20 specimens having a higher number of affected bones in the left limb (compare Figures S1 and S2). In contrast, only 5 specimens out of 20 had a higher number of affected bones in the right limb. The difference was statistically significant (Wilcoxon signed-rank test, *p* value = .007358).

Distinct types of pathologies were detected with higher predominancy on the left side. *μ*CT scan analysis showed that syndactylies were predominantly found in left forelimbs (6 cases) in contrast to the right (2 cases). There were no sex differences in their appearance. Three males and 3 females were affected. Similarly, cleft of the autopodium was present in 9 left and only in 5 right forelimbs. The left predominancy was observed also in sesamoid bones abnormalities (13 left and 8 right forelimbs). Interestingly, no sex differences were found in their appearance similarly to the syndactylies (7 males and 6 females). In contrast, camptodactyly was observed in 7 females compared to only 4 males. Interestingly, synostoses were present independent of side and sex in every affected specimen and in every affected forelimb.

The left-sided predominancy was observed in the metacarpal area pathologies, thus the metacarpal bones of the left autopodium evinced higher frequencies of pathologies in contrast to the right limbs. The number of affected bones was also higher on the left.

Interestingly, the right forelimbs in females were more severely affected than in males (Table 5, compare Figures S1 and S2). In general, the left forelimbs were more affected than the right. In males, the left forelimbs evinced significantly higher severity (higher number of bones with pathologies) compared to the right (Wilcoxon signed-rank test, *p* value = .03175). The difference between the left and right forelimbs in females was not significant (Wilcoxon signed-rank test, *p* value = .1602). Distinct types of pathologies were detected in both males and females and cumulatively with left predominancy. However, when observing laterality differences in both sexes separately than the left predominancy was cardinal only for males. This applied for sesamoid bone defects where the males had affected 7 left and 3 right forelimbs but females 6 left and 5 right forelimbs. A similar situation was observed with the cleft pathology, where in males 5 left and 2 affected right forelimbs were observed whereas in females 4 left and 3 right forelimbs were found.

**Table 5:** Distribution of defects in postnatal *Spry2+/−;Spry4−/−* according to sex and laterality.

Affected specimen count					Forelimb count											
	Male		Female		Male		Female		Male left		Male right		Female left		Female right	
**Total, *n* (%)**	9	(100)	10	(100)	17	(100)	20	(100)	9	(100)	8	(100)	10	(100)	10	(100)
**Syndactyly, *n* (%)**	3	(33)	3	(30)	4	(24)	4	(20)	3	(33)	1	(13)	3	(30)	1	(10)
**Oligodactyly, *n* (%)**	6	(67)	1	(10)	6	(35)	1	(5)	6	(67)	0	(0)	1	(10)	0	(0)
**Camptodactyly, *n* (%)**	4	(44)	7	(70)	5	(29)	10	(50)	3	(33)	2	(25)	4	(40)	6	(60)
**Sezamoid, *n* (%)**	7	(78)	7	(70)	10	(59)	11	(55)	7	(78)	3	(38)	6	(60)	5	(50)
**Cleft, *n* (%)**	5	(56)	4	(40)	7	(41)	7	(35)	5	(56)	2	(25)	4	(40)	3	(30)
**Nodules, *n* (%)**	3	(33)	5	(50)	4	(24)	7	(35)	3	(33)	1	(13)	4	(40)	3	(30)
**Synostosis, *n* (%)**	9	(100)	10	(100)	17	(100)	18	(90)	9	(100)	8	(100)	9	(90)	9	(90)

Nine out of 9 males and 10 out of 11 females were affected. One *Spry2+/−;Spry4−/−* female did not present any limb pathology.

Distinct pathologies showed sex and laterality differences (Table 5). Camptodactyly was detected in 7 females and only 4 males with 10 forelimbs affected in females and 5 forelimbs in males. Moreover, in females 6 right forelimbs were affected in contrast to only 4 left ones. In males, there was no difference between the laterality of affected forelimbs (3 left and 2 right forelimbs). Similarly, ectopic bony nodules were present in 5 females and 3 males (7 female and 4 male forelimbs). Interestingly, in males, the left forelimbs were affected more frequently than the right ones (3 left and 1 right forelimb), and in females, the frequency was more or less equal (4 left and 3 right forelimbs).

### *Sprouty2* and *Sprouty4* have a dynamic and complementary expression pattern during normal autopodium development

**Figure 3:**
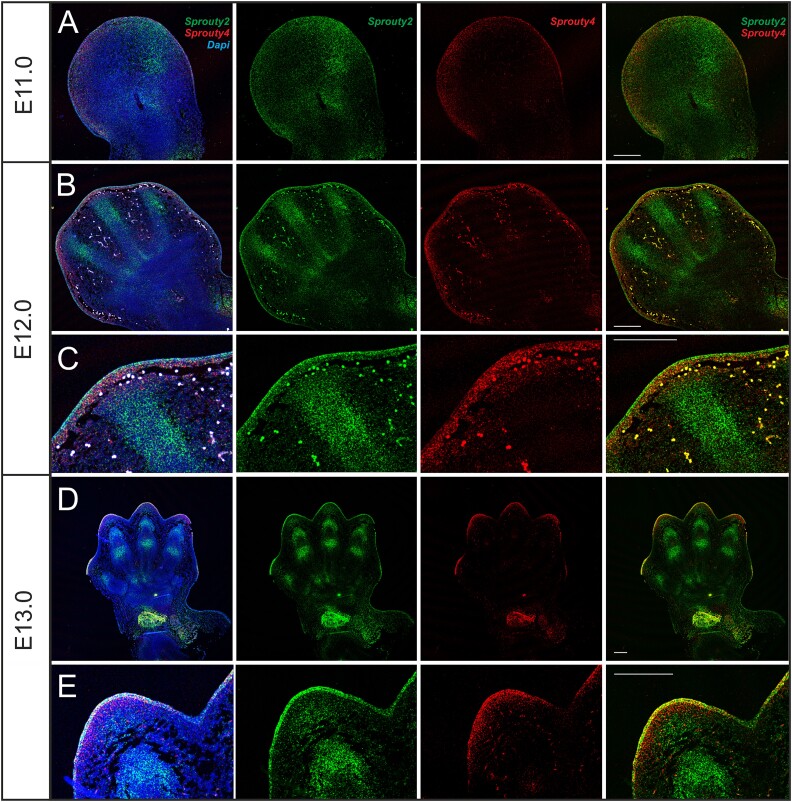
*Sprouty2* and *4* expressions in WT (CD1) mice forelimbs visualized using RNAscope from E11.0 till 13.0. At E11.0, *Sprouty2* was expressed in the posterior area of the developing autopodium. *Sprouty4* expression was limited to the marginal zone of the limb bud (A). At E12.0, the expression of *Sprouty2* was limited to the developing digits and prospective metacarpal area. Interdigital spaces were *Sprouty2* negative. *Sprouty4* expression remained detectable in the marginal zone of the developing autopodium (B, C). At E13.0, *Sprouty2* expression was detectable in the prospective phalangeal bones as well as in the developing carpal area. *Sprouty4* was expressed at the margins of the developing fingers and in the carpal area (D, E). Bar = 200 μm.

*Sprouty2* and *4* have been shown to overlap during early limb bud formation at E11.5.^8^^,^^14^ Coexpression of *Sprouty2* and *4* was, therefore, followed by RNAscope on sections from E11.0 to E13.0 in WT embryos (Figures 3 and S3). At E11.0, *Sprouty2* was expressed in the distal and posterior area of the developing autopodium. It extended from the superficial epithelial lining to the posterior area of the forelimb mesenchyme. *Sprouty4* expression was limited to the marginal zone of the limb bud with a complementary expression to *Sprouty2* (Figure 3A). At E12.0, the expression of *Sprouty2* was limited to the developing digits and prospective metacarpal area. Interdigital spaces were *Sprouty2* negative (Figure 3B, C). *Sprouty4* was observed in the tissue directly under the AER (Figure 3C). By E13.0, *Sprouty2* expression was detectable in the area of the prospective phalangeal bones as well as in the developing carpal area (Figure 3D). *Sprouty4* was expressed in the epithelium at the margins of the developing fingers and in the mesenchyme of the carpal area (Figure 3E). Overlap of *Sprouty2* and *4* was, therefore, concentrated to the carpal area at later stages, and in the distal mesenchyme around the forming digits. The expression pattern of *Sprouty2* was also analyzed by WISH from E12.5 to E16.5 in both the fore and hindlimbs (Figure S4). The early expression in the carpal area mirrored the signal observed in section, with expression extending to the digits at later stages. Interestingly, the expression detected in the forelimbs and hindlimbs showed a very similar pattern, with high levels in the carpal region moving to the digits. The lack of a hindlimb phenotype in the mutants was, therefore, not related to differential expressions of *Sprouty2* in the forelimbs and hindlimbs.

### Disrupted development of the forelimb was apparent from E12 prenatally in *Spry2+/−;Spry4−/−* embryos

**Figure 4:**
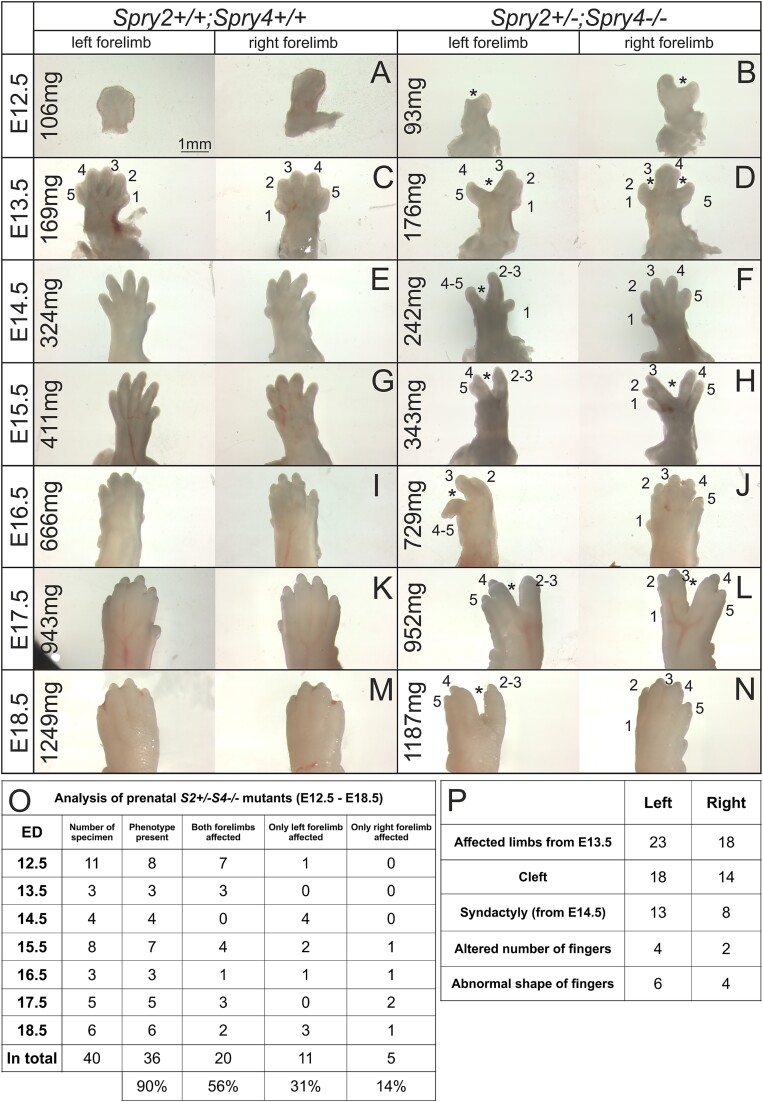
Forelimb pathologies in prenatal *Spry2+/−;Spry4−/−* specimens: E12.5 till E18.5. Compared to control forelimbs (A, C, E, G, I, K, M), *Spry2+/−;Spry4−/−* forelimbs showed a variety of pathologies (B, D, F, H, J, L, M). Earlier than E12.5 pathologies were less clearly detectable in the autopodium. Prenatal analysis was performed from E12.5 to catch 100% of pathologies. The most frequent anomaly was the cleft hand—asterisk, which could be observed unilaterally (left sided in F, J, N) or bilaterally (B, D, H, L). Oligodactyly (on the left-sided forelimb in H, N), polydactyly (on the right side in D) and/or syndactyly (on the left-sided forelimb in F, H, L, N) was detected from E14.5. Quantification of prenatal forelimb pathologies (O, P). The order of fingers is determined for better orientation. The BW of the specimen is stated in milligrams (mg). Scale length is in millimeters (mm).

In order to explain the underlying mechanisms behind the pathologies in the autopodium area in *Spry2+/−;Spry4−/−* animals, we examined prenatal specimens from E12.5 till 18.5 (Table 1, Figures 4 and S5) (*n* = 40). Defects in the forelimbs were detected from E12.5 onwards with a variety of pathologies noted (Figure 4O and P).

In accordance with the postnatal analysis, forelimb pathologies were detected in almost all harvested prenatal specimens (36 out of 40 mutants = 90%). In 56% of the prenatal *Spry2+/−;Spry4−/−* specimens, both forelimbs were affected (Figure 4O). *μ*CT highlighted a higher incidence of defects postnatally, suggesting that additional defects might develop after birth, or might be too subtle to be detected at earlier stages. No significant predominancy for the left sided pathologies was observed in the prenatal harvested specimens with the left autopodium affected in 31 out of 40 cases and for the right side in 25 out of 40 cases (Figure 4O and P).

The most frequent prenatal defect was a cleft of the autopodium (20 out of 25 affected right forelimbs, 23 out of 31 affected left forelimbs) (Figure 4B, D, F, H, J, L, and N). As with the postnatal analysis, the left forelimb was more severely affected than the right. From E13.5 onwards, 78% of autopodium with a cleft on the left side showed additional pathologies (14 out of 18 autopodia with clefts) compared to only 33% of right forelimbs (5 out of 15). Additional defects frequently included syndactyly (10 cases out of 13 totally detected syndactylies were related to a cleft). An abnormal number of fingers (oligodactyly/polydactyly) was present in 3 out of 4 detected cases in relation to left sided clefts (eg, Figure 4F and H). Abnormal shape of the digits was associated with a cleft in 3 out of 6 detected cases. Interestingly, in 3 cases syndactyly was present together with alternations to finger number.

Interestingly, only 7 forelimbs out of 43 forelimbs with cleft of the autopodium evinced cleft on the anterior side of the autopodium (Figure 4D). In the majority of forelimbs with cleft (30 out of 43), the cleft was located more posteriorly (Figure 4D, H, J). In 3 cases, the cleft was associated with an abnormal number of fingers in the midline, and in 3 cases, the limbs had 2 clefts present (Figure 4D right limb). This posterior dominance is in keeping with the more posterior expression domain of *Sprouty2* at E11.0 (Figure 3A).

### Early induction of prospective skeletal patterning defects in Sprouty deficient embryos was related to abnormal formation of the AER

**Figure 5:**
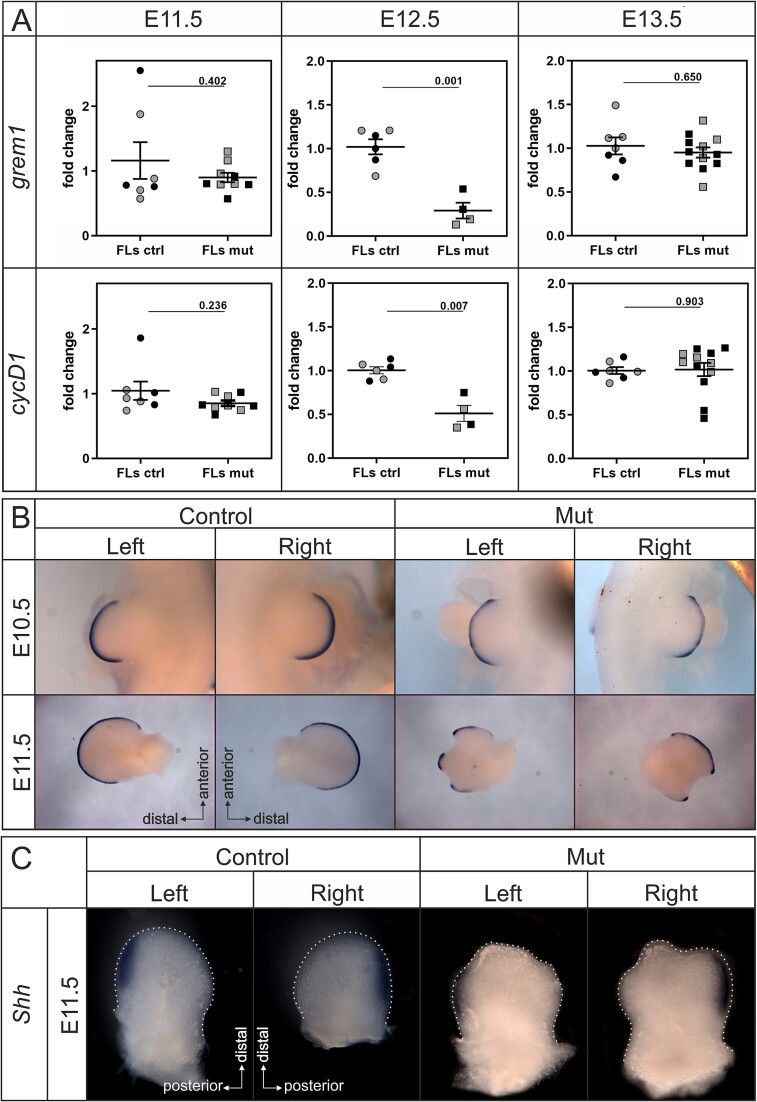
Defects in limb signaling factors in Sprouty deficient embryos. qPCR of *Sprouty2/4* deficient embryos E11.5-13.5 (A). Combinations of Sprouty alleles: *Sprouty2+/+;Sprouty4+/+*, *Sprouty2+/-;Sprouty4+/+* and *Sprouty2-/-;Sprouty4+/+* are referred to as controls, ctrl (20 forelimbs in total) and embryos with combinations of Sprouty alleles: *Sprouty2+/+;Sprouty4-/-*, *Sprouty2+/-;Sprouty4-/-* and *Sprouty2-/-;Sprouty4-/-* are referred to as mutants, mut (25 forelimbs in total). Relative fold change in gene expression was calculated using 2^*−*∆∆Ct^ method and plotted on the graph. Both left (gray data sets) and right (black data sets) limbs were included in the analysis. For gene expression calculation, each left/right mutant forelimb was related to a corresponding left/right control forelimb. *Fgf8* expression in forelimbs of control and mutant embryos (Mut) at E10.5-11.5 visualized using WISH (B) *Spry2+/+;Spry4+/+* and *Spry2+/−;Spry4−/−* at E10.5 and *Spry2+/−;Spry4+/+* and *Spry2+/−;Spry4−/−* embryos at E11.5 were used for *Fgf8* ISH. At E10.5-11.5, *Fgf8* was expressed in the area of the apical ectodermal ridge of the developing autopodium of both left and right forelimbs in control specimens. In *Sprouty* deficient embryos, the area of *Fgf8* expression was slightly weaker and it was discontinuous in the forelimbs autopodium at E10.5 and interrupted in both left and right autopodia at E11.5 reflecting the appearance of cleft. (C) *Shh* expression in the posterior region (ZPA) of the forelimbs was not altered in *Spry2+/−;Spry4−/−* embryos at E11.5 despite signs of cleft formation in the mutants. Abbreviations: ZPA, zone of polarizing activity.

In order to determine the effect of Sprouty2/4 deficiency on proliferation, qPCR analysis was performed for *CycD1*, an important regulator of the cell cycle, and *Grem1*, which acts in a Shh/Grem1/Fgf epithelial-mesenchyme feedback system to control outgrowth.^27^ Due to differences in BW at these early stages, and expected variability in the Sprouty deficient samples, differences in proliferation rate were difficult to observe by immunofluorescence; therefore, samples were pooled for analysis by qPCR. qPCR revealed significant differences between the *Sprouty2/4* deficient embryos and control forelimbs at E12.5 with downregulation of both *CycD1* and *Grem1*, although no difference was noted at E11.5 and E13.5 (Figure 5A). In keeping with the change at E12.5, this was the first stage where the cleft of the autopodium was clearly detectable in the *Spry2+/−;Spry4−/−* limb bud (Figure 4B). Downregulation of *CycD1* at E12.5 suggested a reduced proliferation and *Grem1* downregulation at the same stage suggested a dysregulation of the Shh/Fgf feedback loop in the early limb bud. To investigate the potential defect on limb signaling centers the AER was investigated using expression of *Fgf8* as a marker (Figure 5B). In control embryos, *Fgf8* was expressed along the AER in the developing autopodium of both left and right forelimbs. However, in *Spry2+/−;Spry4−/−* forelimbs *Fgf8* expression was interrupted along both left and right autopodia (Figure 5B) (*n* = 2). Importantly, regions with loss of *Fgf8* corresponded to the formation of clefts, where the integrity of the AER was disrupted. Such breaks in the AER can, therefore, explain the autopodium clefts observed at later stages.

### Altered contribution of the cells of the ZPA to the limb

Given that anterior-posterior abnormalities were frequently observed postnatally, changes to the ZPA were also assessed. For this, the expression of *Shh* was analyzed in the mutant at E11.5. Expression of *Shh* was unchanged in the mutant, despite the mutant limbs displaying early signs of defects (Figure 5C) suggesting that initiation of the ZPA was not affected in the mutant.

**Figure 6:**
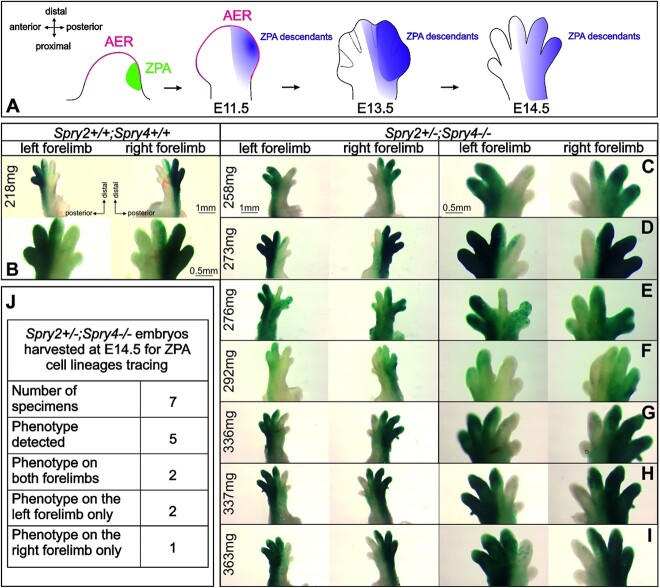
Altered distribution of Shh descendant cells in *Spry2+/−;Spry4−/−* specimens. (A) Schematic showing the normal distribution of cells active in the ZPA (*Shh* expression in green) and AER (*Fgf8* expression in red) during early development. Distribution of ZPA descendants visualized using X-gal staining in Shh reporter mice from E11.5 to E14.5. (B) In control forelimbs at E14.5 positive cells were detectable in the area of prospective digit 4 and 5 and up to the midline of the third digit. (C-I) In *Spry2+/−;Spry4−/−* specimens positive cells were present in the anterior regions of the forelimbs at E14.5, documenting abnormal migration of the ZPA cells. (J) Distribution of pathologies in *Spry2+/−;Spry4-/−* specimens used for cell lineage tracings. The BW of the specimens is stated in milligrams (mg). Scale length is in millimeters (mm). Abbreviations: AER, apical ectodermal ridge; ZPA, zone of polarizing activity.

Although no effect on the expression of *Shh* was observed at E11.5, we then assessed the contribution of the *Shh* expressing cells of the ZPA to the limb (Figure 6). Limbs were harvested from embryos derived from the crosses between *Sprouty2/4*/*Shh*LacZ females mated to *Sprouty2/4/Shh*/EGFP/Cre males and were stained for LacZ. In control limbs, the ZPA descendants contributed to the posterior two most fingers, with contribution to the posterior half of the middle finger, and a sharp boundary with the anterior limb, which showed no descendant cells (Figure 6A and B), agreeing with previous lineage analysis.^21^ In mutant limbs (*n* = 7 at E14.5), abnormal migration of cells was observed in the anterior zone of the autopodium, which is normally *Shh* negative (Figure 6C-I). Interestingly, in *Spry2+/−;Spry4−/−* forelimbs with midline clefts, only the 2 most posterior digits were descended from the ZPA, with the anterior part of the fourth digit unlabeled (Figure 6C and F). The specimens with other defects (such as abnormal or partially fused fingers) evinced ectopically present positive cells in the anterior region of the limb when compared to controls (Figure 6E and I). The distinct lineage restriction of the ZPA cells to the posterior of the limb was, therefore, compromised in the mutant, this effect being even more prominent in double knockouts (*Spry2−/−;Spry4−/−)* (Figure S5).

## Discussion

*Spry2+/−;Spry4−/−* mice displayed a wide range of limb defects impacting all regions of the limb. The mildest defects, such as the presence of synostoses, were only detectable by using *μ*CT scans. The *μ*CT analysis revealed defects in almost 100% of postnatal *Spry2+/−;Spry4−/−* specimens compared to approximately 50% detected by visual inspection. The number of limbs with recorded pathologies postnatally was higher than prenatally, highlighting the accuracy of the *μ*CT imaging technique and the increase in more minor pathologies during later limb development. The range of defects reflected the dynamic expression of *Sprouty2* and *4* during limb development, with Sproutys playing several roles at different stages.

Previous data analysis using *Sprouty4* null mice have shown polydactylies in combination with syndactylies at 100% penetrance in forelimbs.^16^ In contrast, the *Spry2+/+;Spry4−/−* in our colony had a very low penetrance of defects (data not shown). This is likely to reflect different genetic backgrounds. In our colony, loss of *Sprouty4* only led to an impact on forelimb development when combined with *Sprouty2* deficiency, suggesting some compensatory mechanism acting between Sprouty*2* and *4*. This was despite these two Sproutys showing largely complementary expression at early stages of limb development.

Our study highlights the variability of the phenotype in the forelimb defects in *Spry2+/−;Spry4−/−* mice. Some mice had mild phenotypes, only evident when revealed by *μ*CT scanning postnatally, while others had dramatic clefts and digit changes. Variable phenotypes and severity of abnormalities in Sprouty2/4 deficiency have also been described in other organs.^19^ This variability may be due to the presence of one copy of *Sprouty2* in our *Spry2+/−;Spry4−/−* analysis. Phenotypic variation has been shown to be explained by a nonlinear relationship between gene expression and morphological outcome.^28^ Using this model, differences in robustness to perturbation are not caused by gene expression variance or dysregulation but due to this nonlinearity of the genotype-phenotype curve.

In specimens where both forelimbs were affected, the left limb had a stronger phenotype compared to the right. This was most clearly observed with the clefts of the forelimb, where clefts were predominantly on the left in the postnatal samples, and a right sided cleft was only present in combination with a left side cleft. Interestingly, left-biased limb defects were detected in Holt-Oram syndrome patients,^29^ while mice with *Fgf8* deficiency also showed a stronger defect on the left side.^30^ In organs with left-right symmetrical development, such as limbs, there are slight left-right differences in their molecular regulation.^31^ The left-right asymmetry of early embryo is determined very early during development. The cells of primitive streak and primitive node express *Fgf8*, which induces *Nodal* expression on the left side of the embryo.^32^ The rotation movements of cilia in the primitive node lead to the movement of fluid to the left to establish this asymmetry in mouse embryos.^33^ Abnormally elongated primary cilia have been detected recently in *Sprouty2* and/or *Sprouty4* deficient mice, and the phenotype of these animals has been related to ciliopathies.^19^ The higher penetrance of severe limb defects on the left in the *Spry2+/−;Spry4−/−* mice here, therefore, may result from abnormal ciliary function in the early embryo.

Interestingly, the hindlimbs were normally developed in all *Spry2+/−;Spry4−/−* specimens in our study. This was despite there being no obvious difference in expression of *Sprouty2*. The differencial response in limbs, therefore, will require further analysis.

The clefts and digit specification defects were identified early in embryonic development. Here, we focused on the mechanisms that drove these dramatic early defects by investigating the impact of reduction of Sproutys on the AER and ZPA. During normal development, *Sprouty4* was expressed in the mesenchyme adjacent to AER of the limb bud, while *Sprouty2* was expressed early on in the posterior region near the ZPA. AER abnormalities appear very early during development and thus can be considered as the main initiation factors for autopodium pathologies.^34^  *Ectrodactyly*, split hand, or cleft hand in patients involves the deficiency or absence of one or more central digits of the hand and its presence has been related to the alterations in AER^35^ as well as to the defects in the feedback loop between the ZPA and AER.^36^ In our *Spry2+/−;Spry4−/−* mice, we observed abnormal AER formation at E11.5 with disrupted expression of *Fgf8*. Importantly, in the regions where *Fgf8* expression was missing, the underlying tissue was reduced suggesting alteration of proliferation. An alteration of proliferation (reduced *CyclinD1*) was also evident using qPCR. Interestingly, we did not observe a downregulation of *CyclinD1* at E13.5, potentially suggesting the tissue had managed to restore levels of proliferation by this stage. *Gremlin 1* was also downregulated at E12.5. *Gremlin1* acts in feedback loop involving Shh from the ZPA and Fgf8 from the AER, and, therefore, downregulation suggested a loss of interaction between these two signaling centers when Sprouty levels are reduced. Downregulation of *Shh* expression has been observed previously in specimens with postaxial ectrodactyly in forelimbs.^37^ In this case, the lower levels of Shh were related to abnormal apoptosis in the mesenchyme of forelimb autopodia leading to cleft formation. In our *Spry2+/−;Spry4−/−* mice, however, the expression of *Shh* in early limbs appeared unaffected. Interestingly, however, the contribution of the *Shh* expressing ZPA cells to the limb was impacted in the mutants. In WT mice, the *Shh* expressing ZPA cells only contribute to the posterior half of the limb.^21^ In our *LacZ/ShhEGFPCre* embryos, a very clear midline boundary was observed along the middle finger in control WT mice. In contrast, in the *Spry2+/−;Spry4−/−* mice, *Shh* descendent cells were often found on the anterior side of the limb. The normal restriction of these cells to one side was therefore disrupted by *Sprouty* deficiency. The clefts and finger patterning defects observed in the *Spry2+/−;Spry4−/−* mice were likely to be caused by a combination of loss of integrity of the AER (clefts), combined with a loss of anterior-posterior identity (digit defects).

The loss of Fgf8 in the AER and impact on the fate of ZPA cells may be driven by enhanced FGF signaling in the limbs,^38^ or due to the effect of loss of Sproutys on primary cilia,^19^ given that primary cilia are regulators of both Shh and FGF signaling pathways.^39^^,^^40^ Interestingly, changes in the length of the primary cilia, due to disrupted FGF signaling, correlated with changes in Shh response.^41^

From E13.5, *Sprouty2* expression moved to the prospective phalangeal bones of the digits. The later defects in the limb noted in *Spry2+/−;Spry4−/−* mice may also be explained by changes to the primary cilia. Skeletal dysplasia, shortening of limb long bones, and polydactyly in humans is a frequent phenotype related to ciliopathies.^42^ Elongated primary cilia were observed in the chondrocytes of growth plates in *Sprouty2/4* deficient mice, in both embryos and postnatal animals.^19^ Primary cilia are important for chondrocyte and osteoblast differentiation, as well as for the function of osteocytes.^43^^,^^44^ The abnormal ossification, observed as frequent synostoses in the limbs of *Spry2+/−;Spry4−/−* mice, could, therefore, be explained by disrupted differentiation of these cells.

Interestingly, camptodactyly was detected postnatally in 41% of our samples. Camptodactyly has previously been linked to disrupted *Fgfr3* expression.^45^  *Sprouty* deficiency may, therefore, also provide an understanding of the mechanisms underlying findings in these Fgf mutants.

*Sprouty2* expression has also been associated with muscles and tendon differentiation in the chick and mouse limb development.^15^ Sproutys have been shown to participate in the physiological muscle development via their direct involvement in the process of myogenic differentiation^46^ or via preventing the progenitors from entering the skeletal differentiation program.^47^ The *Spry2+/−;Spry4−/−* mice may therefore also exhibit muscle defects not picked up by our *μ*CT and skeletal preparation analysis.

In conclusion, loss of *Sprouty4* with loss of one copy of *Sprouty2* led to complex forelimb pathologies, the most striking of which was the formation of autopodium clefts. These clefts formed early in limb bud formation linked to loss of FGF signaling in the AER. Loss of Fgf8 was associated with downregulation of proliferation markers and of *Gremlin1*, highlighting defects in AER/ZPA interactions. *Shh* expression was not obviously impacted in the *Spry2+/−;Spry4−/−* mice, but the descendants of *Shh* expressing cells were no longer constrained to the posterior half of the forming limb. The AER and ZPA changes are likely to explain many of the pathologies associated with fused and supernumerary digits, while later role of Sproutys in chondrogenesis and osteogenesis explain the later limb defects. These findings can help our understanding of limb defects in patients, particular those with complex etiology.

## Supplementary Material

Supplementary_Figure_1_ziaf002

Supplementary_Figure_2_ziaf002

Supplementary_Figure_3_ziaf002

Supplementary_Figure_4_ziaf002

Supplementary_Figure_5_ziaf002

Supplementary_figures_ziaf002

## Data Availability

The data that support the findings of this study are available in the supplementary material of this article.
