# Supplementary material for: Sprouty2/4 deficiency disrupts early signaling centers impacting chondrogenesis in the mouse forelimb
Source: JBMR Plus. 2025 Jan 10;9(3):ziaf002. doi: 10.1093/jbmrpl/ziaf002 (PMC11792080; doi:10.1093/jbmrpl/ziaf002)
Supplement: Supplementary_Figure_1_ziaf002 [file supplementary_figure_1_ziaf002.pdf]

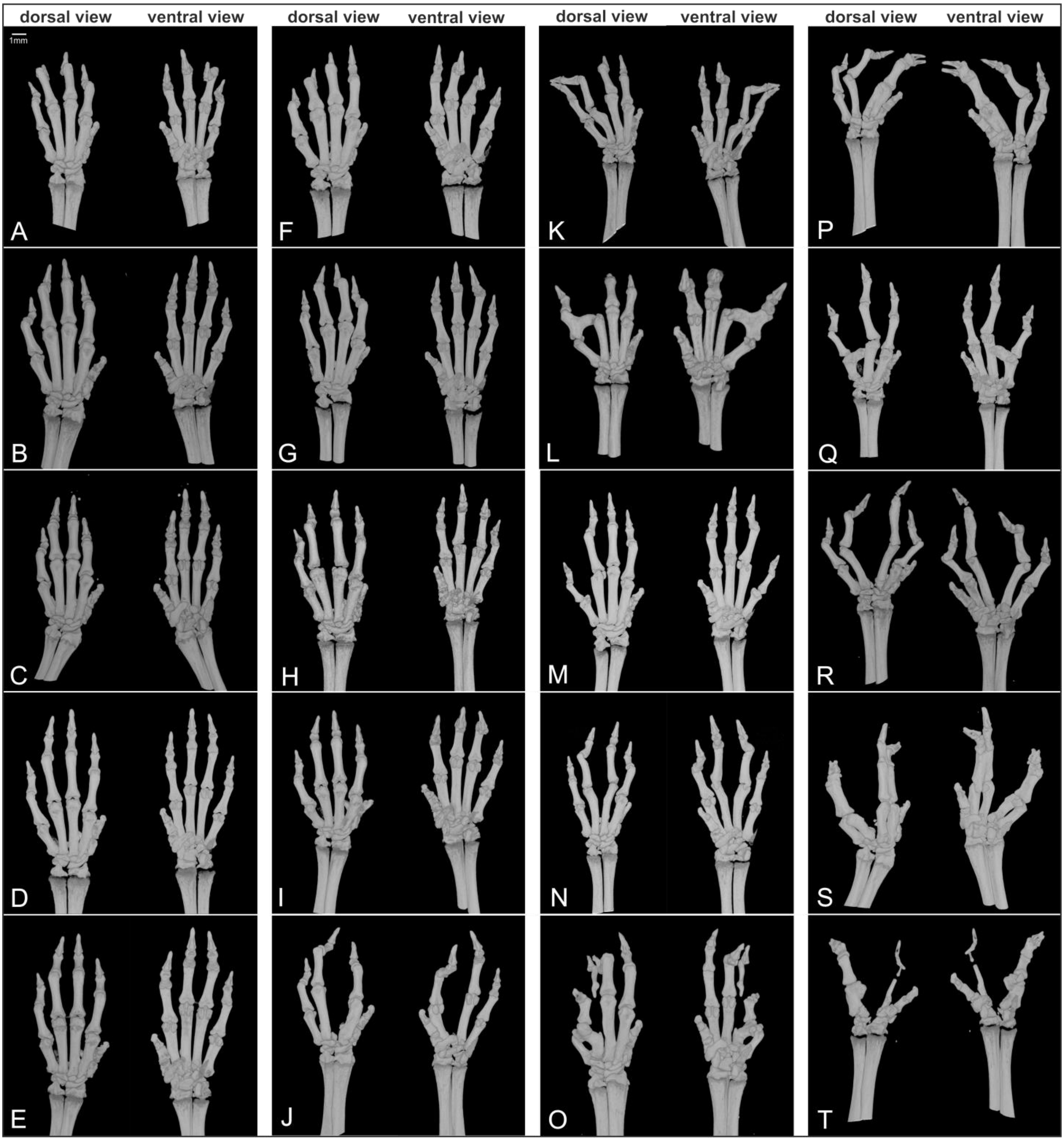

S1: *μ*CT scans of the left autopodium of adult specimens *Spry2+/-;Spry4-/-.*

The number of affected bones per limb (the severity of affection) increases from a physiological state in A to T. The left limb is more severely affected in 70% of all *Spry2+/-;Spry4-/-* mice. The number of affected bones per limb is in average 2 times higher in the left forelimbs than in the right ones.
