# Supplementary material for: Sprouty2/4 deficiency disrupts early signaling centers impacting chondrogenesis in the mouse forelimb
Source: JBMR Plus. 2025 Jan 10;9(3):ziaf002. doi: 10.1093/jbmrpl/ziaf002 (PMC11792080; doi:10.1093/jbmrpl/ziaf002)
Supplement: Supplementary_Figure_2_ziaf002 [file supplementary_figure_2_ziaf002.pdf]

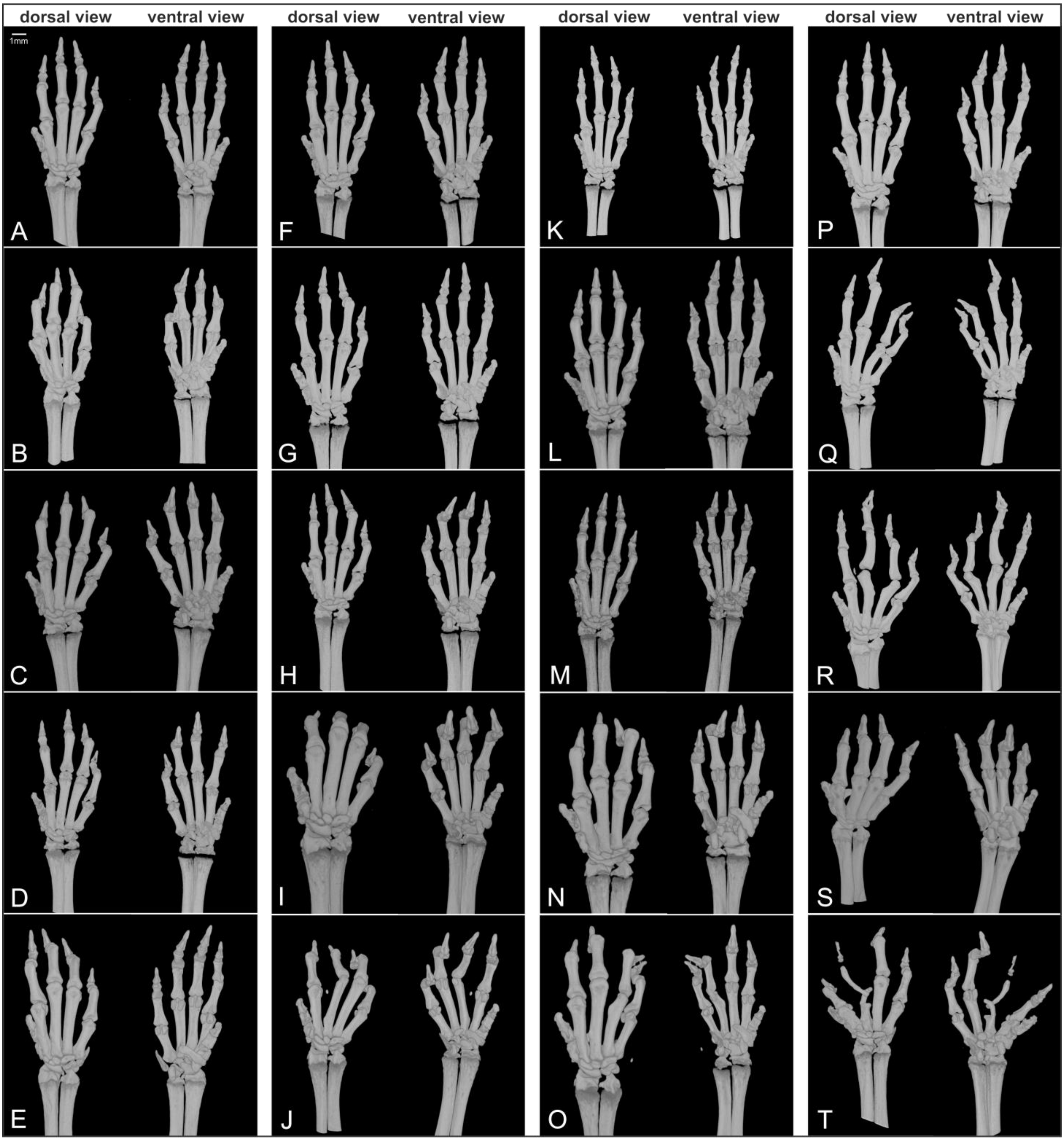

S2: μCT scans of the right autopodium of adult specimens Spry2+/-;Spry4-/-.
The number of affected bones per limb (the severity of affection) increases from a physiological state in A to T. Interestingly, the right forelimbs in females (B, C, E, F, L, N, P, Q, R, S, T) are more severely affected than in males (A, D, G, H, I, J, K, M, O).
