# Supplementary material for: Sprouty2/4 deficiency disrupts early signaling centers impacting chondrogenesis in the mouse forelimb
Source: JBMR Plus. 2025 Jan 10;9(3):ziaf002. doi: 10.1093/jbmrpl/ziaf002 (PMC11792080; doi:10.1093/jbmrpl/ziaf002)
Supplement: Supplementary_Figure_3_ziaf002 [file supplementary_figure_3_ziaf002.pdf]

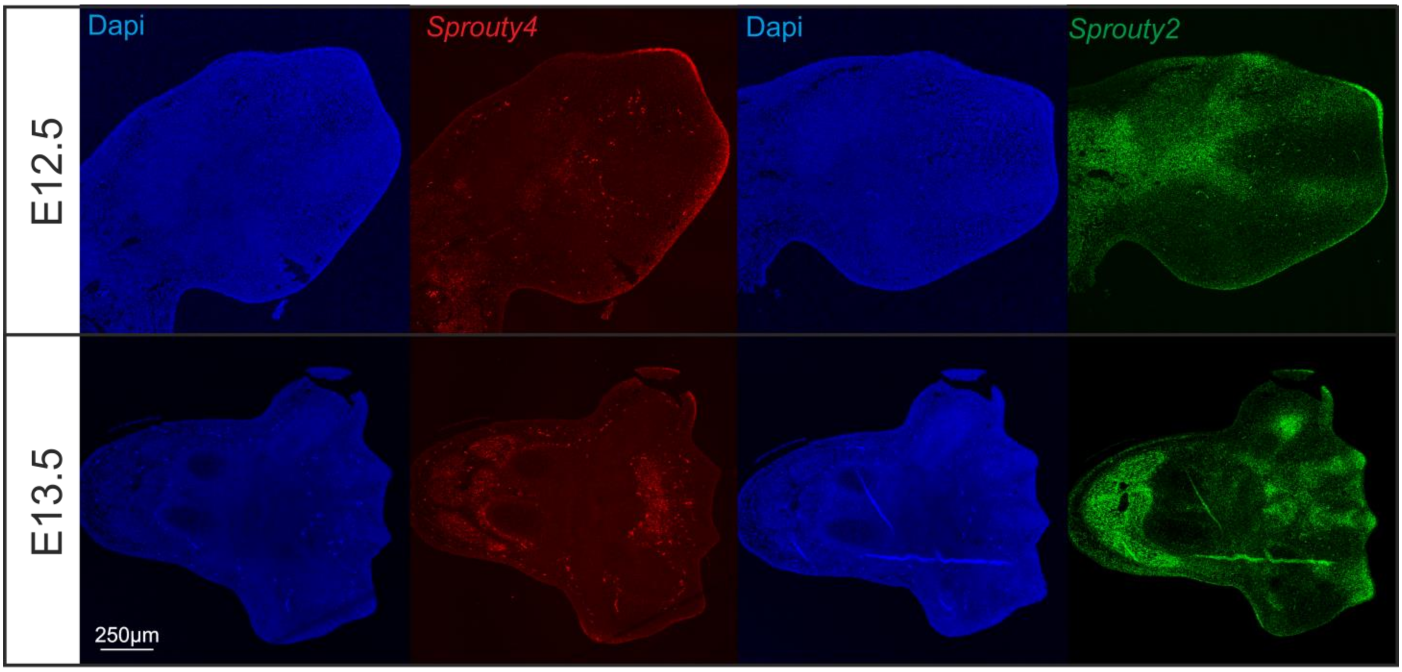

S3: *Sprouty2* and *Sprouty4* expressions in WT (B6) mouse forelimbs visualized using RNAscope at E12.5 and 13.5.
At E12.5, the expression of *Sprouty2* is limited to the developing digits and prospective metacarpal area. Interdigital spaces are *Sprouty2* negative. *Sprouty4* expression is detectable in the marginal zone of the developing autopodium. At E13.5, *Sprouty2* expression is detectable in the prospective phalangeal bones as well as in the developing carpal area. *Sprouty4* is expressed at the margins of developing fingers and in the carpal area. The photographs document same expression patterns as observed in CD1 embryos shown in Fig. 3.
