# Supplementary material for: Sprouty2/4 deficiency disrupts early signaling centers impacting chondrogenesis in the mouse forelimb
Source: JBMR Plus. 2025 Jan 10;9(3):ziaf002. doi: 10.1093/jbmrpl/ziaf002 (PMC11792080; doi:10.1093/jbmrpl/ziaf002)
Supplement: Supplementary_Figure_4_ziaf002 [file supplementary_figure_4_ziaf002.pdf]

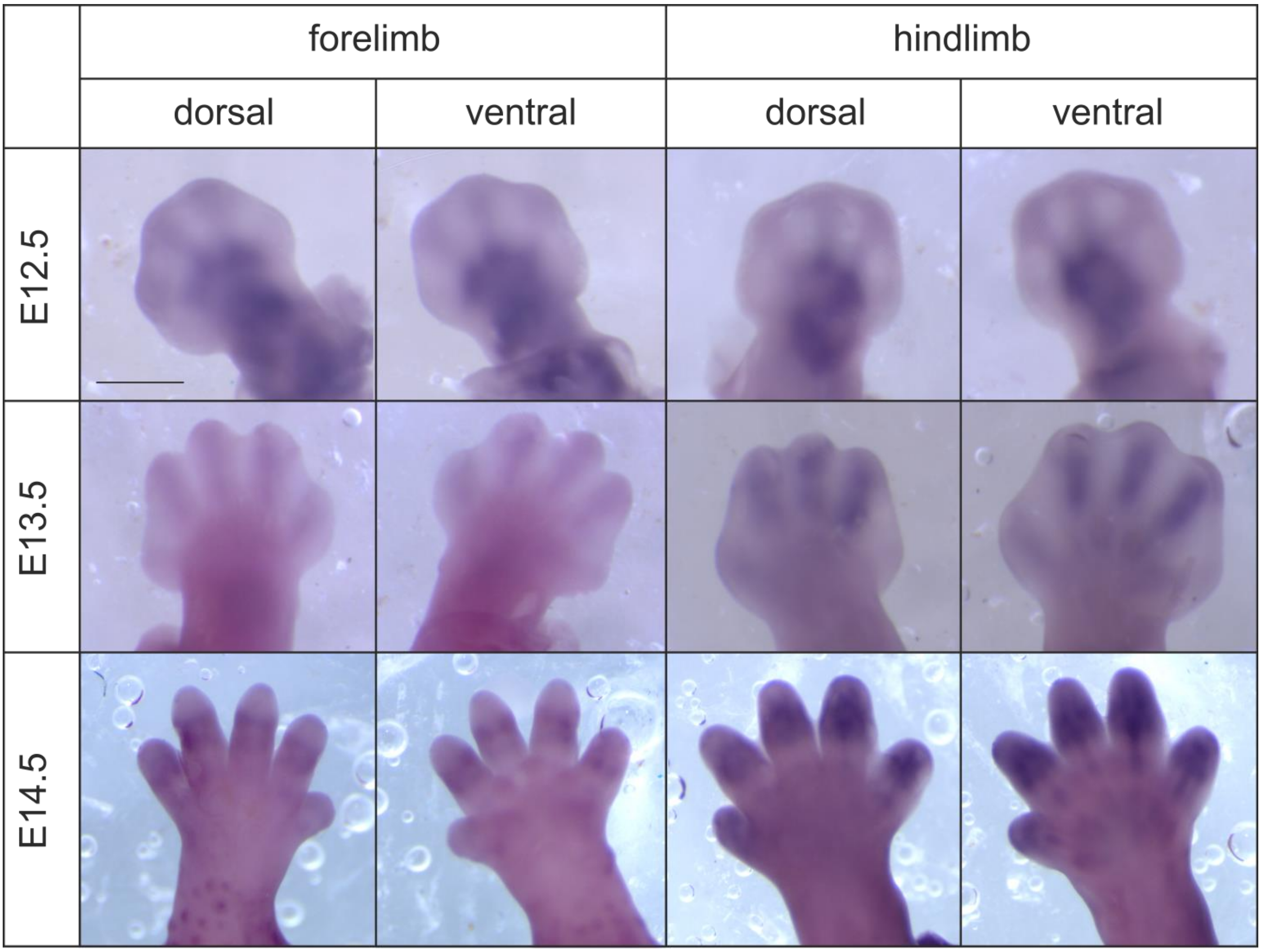

S4: *Sprouty2* expression in fore- and hindlimbs of WT mice visualized using *in situ* hybridization from E12.5 till 14.5.

At E12.5 *Sprouty2* is expressed in the prospective carpal and metacarpal area of the developing autopodium and in the area of prospective long bones. At E13.5, the expression is limited to the developing digits. Interdigital spaces in all specimens are *Sprouty2* negative. At E14.5, *Sprouty2* expression is detectable in the area of prospective phalangeal bones. Interestingly, the expressions detected in the forelimbs and hindlimbs show very similar patterns. Bar – 1mm. More advanced stages (E15.5 and 16.5) were *Sprouty2* negative.
