# Supplementary material for: Sprouty2/4 deficiency disrupts early signaling centers impacting chondrogenesis in the mouse forelimb
Source: JBMR Plus. 2025 Jan 10;9(3):ziaf002. doi: 10.1093/jbmrpl/ziaf002 (PMC11792080; doi:10.1093/jbmrpl/ziaf002)
Supplement: Supplementary_Figure_5_ziaf002 [file supplementary_figure_5_ziaf002.pdf]

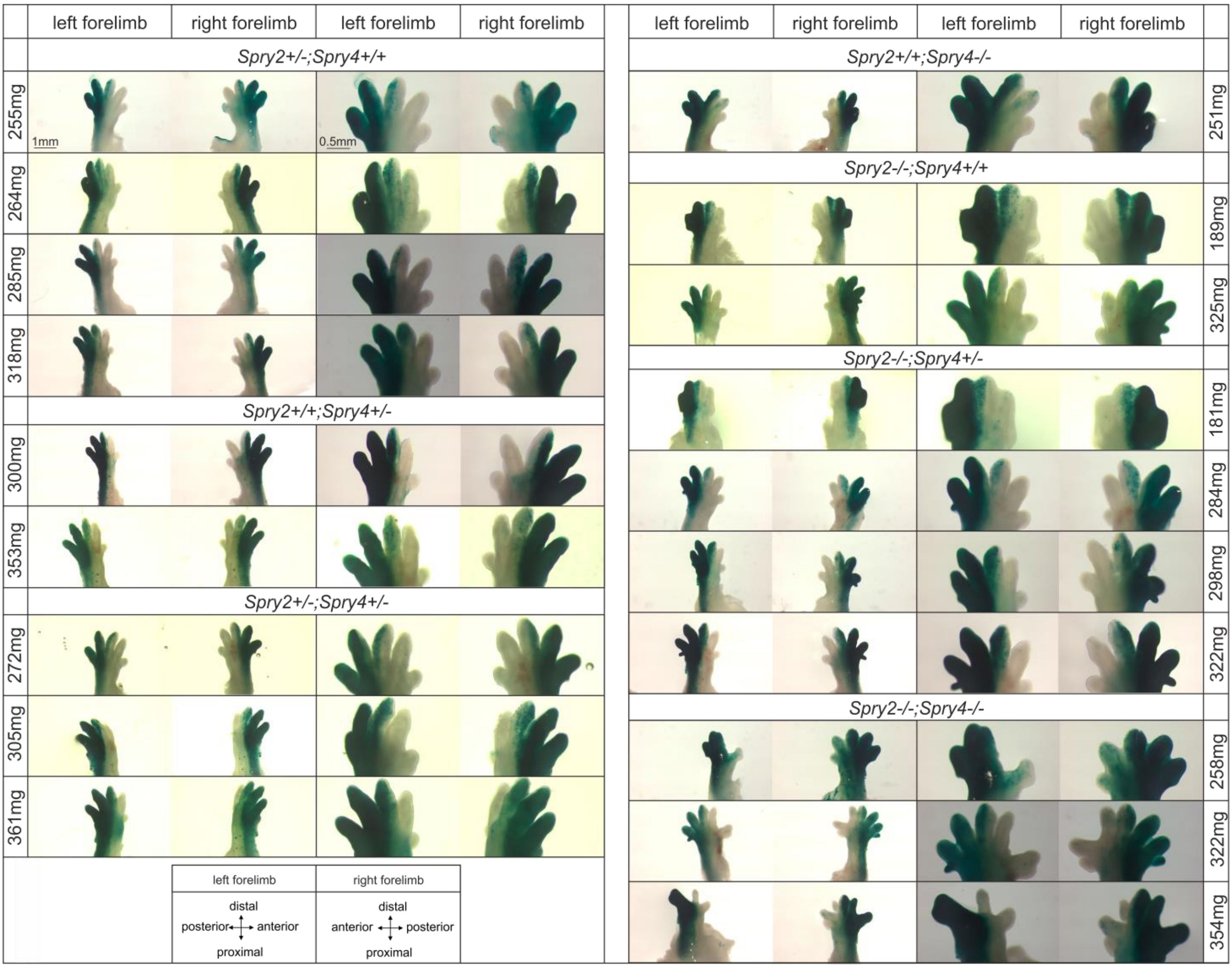

S5: Distribution of ZPA *Shh* expressing descendants visualized using X-gall staining (blue) at E14.5 in embryos with different *Sprouty2* and *Sprouty4* genes dosages.

In the specimens with lower *Sprouty4* dosages positive cells are detectable in the anterior regions of forelimbs documenting abnormal migration of ZPA. This is correlated with higher frequencies of pathologies detected in specimens with lower *Sprouty4* dosages. All specimens with the lowest dosages of *Sprouty2* and *4* genes (*Spry2-/-;Spry4-/-*) evince abnormal migration of ZPA together with abnormal digit numbers and clefts associated with digit fusions.
