# Supplementary material for: Sprouty2/4 deficiency disrupts early signaling centers impacting chondrogenesis in the mouse forelimb
Source: JBMR Plus. 2025 Jan 10;9(3):ziaf002. doi: 10.1093/jbmrpl/ziaf002 (PMC11792080; doi:10.1093/jbmrpl/ziaf002)
Supplement: Supplementary_figures_ziaf002 [file supplementary_figures_ziaf002.docx]

**Supplementary figures:**

**S1**

*µ*CT scans of the left autopodium of adult specimens *Spry2+/-;Spry4-/-*.

The number of affected bones per limb (the severity of affection) increases from a physiological state in A to T. The left limb is more severely affected in 70% of all *Spry2+/-;Spry4-/-* mice. The number of affected bones per limb is in average 2 times higher in the left forelimbs than in the right ones.

**S2**

*µ*CT scans of the right autopodium of adult specimens *Spry2+/-;Spry4-/-*.

The number of affected bones per limb (the severity of affection) increases from a physiological state in A to T. Interestingly, the right forelimbs in females (B, C, E, F, L, N, P, Q, R, S, T) are more severely affected than in males (A, D, G, H, I, J, K, M, O).

**S3**

*Sprouty2* and *Sprouty4* expressions in WT (B6) mouse forelimbs visualized using RNAscope at E12.5 and 13.5.

At E12.5, the expression of *Sprouty2* is limited to the developing digits and prospective metacarpal area. Interdigital spaces are *Sprouty2* negative. *Sprouty4* expression is detectable in the marginal zone of the developing autopodium. At E13.5, *Sprouty2* expression is detectable in the prospective phalangeal bones as well as in the developing carpal area. *Sprouty4* is expressed at the margins of developing fingers and in the carpal area. The photographs document same expression patterns as observed in CD1 embryos shown in Fig. 3.

**S4**

*Sprouty2* expression in fore- and hindlimbs of WT mice visualized using in situ hybridization from E12.5 till 14.5.

At E12.5 *Sprouty2* is expressed in the prospective carpal and metacarpal area of the developing autopodium and in the area of prospective long bones. At E13.5, the expression is limited to the developing digits. Interdigital spaces in all specimens are *Sprouty2* negative. At E14.5, *Sprouty2* expression is detectable in the area of prospective phalangeal bones. Interestingly, the expressions detected in the forelimbs and hindlimbs show very similar patterns. Bar – 1mm. More advanced stages (E15.5 and 16.5) were *Sprouty2* negative.

**S5**

Distribution of ZPA Shh expressing descendants visualized using X-gall staining (blue) at E14.5 in embryos with different *Sprouty2* and *Sprouty4* genes dosages.

In the specimens with lower *Sprouty4* dosages positive cells are detectable in the anterior regions of forelimbs documenting abnormal migration of ZPA. This is correlated with higher frequencies of pathologies detected in specimens with lower *Sprouty4* dosages. All specimens with the lowest dosages of *Sprouty2* and *4* genes (*Spry2-/-;Spry4-/-*) evince abnormal migration of ZPA together with abnormal digit numbers and clefts associated with digit fusions.
